# Bioinspired Stimuli-Responsive Materials for Soft Actuators

**DOI:** 10.3390/biomimetics9030128

**Published:** 2024-02-21

**Authors:** Zhongbao Wang, Yixin Chen, Yuan Ma, Jing Wang

**Affiliations:** 1 State Key Laboratory of Mechanical System and Vibration, School of Mechanical Engineering, Shanghai Jiao Tong University, Shanghai 200240, China; 194106@sjtu.edu.cn (Z.W.); yixin-chen@sjtu.edu.cn (Y.C.); 2 Department of Mechanical Engineering, Research Institute for Intelligent Wearable Systems, The Hong Kong Polytechnic University, Hong Kong 999077, China

**Keywords:** bioinspired actuators, soft robots, stimuli-responsive materials, smart materials

## Abstract

Biological species can walk, swim, fly, jump, and climb with fast response speeds and motion complexity. These remarkable functions are accomplished by means of soft actuation organisms, which are commonly composed of muscle tissue systems. To achieve the creation of their biomimetic artificial counterparts, various biomimetic stimuli-responsive materials have been synthesized and developed in recent decades. They can respond to various external stimuli in the form of structural or morphological transformations by actively or passively converting input energy into mechanical energy. They are the core element of soft actuators for typical smart devices like soft robots, artificial muscles, intelligent sensors and nanogenerators. Significant progress has been made in the development of bioinspired stimuli-responsive materials. However, these materials have not been comprehensively summarized with specific actuation mechanisms in the literature. In this review, we will discuss recent advances in biomimetic stimuli-responsive materials that are instrumental for soft actuators. Firstly, different stimuli-responsive principles for soft actuators are discussed, including fluidic, electrical, thermal, magnetic, light, and chemical stimuli. We further summarize the state-of-the-art stimuli-responsive materials for soft actuators and explore the advantages and disadvantages of using electroactive polymers, magnetic soft composites, photo-thermal responsive polymers, shape memory alloys and other responsive soft materials. Finally, we provide a critical outlook on the field of stimuli-responsive soft actuators and emphasize the challenges in the process of their implementation to various industries.

## 1. Introduction

Biological species can react to environmental stimuli with millisecond motion response, and these responses are mostly generated through soft tissue systems, for example, muscle contraction. From lipid deformation in single-cell bacteria [1] to multi-muscle contraction in butterfly wing motion [2], to the bone–muscle system in mammals [3], nature has provided us with abundant examples of soft actuators. Therefore, we are in an era where complex soft actuation systems are being created, bridging the gap between conventional rigid robotic systems developed during the Industrial Revolution and soft robotics emerging in the last decade.

Inspired by deformable, adaptive and reconfigurable environmental-responsive biomaterial systems in nature, artificial stimuli-responsive materials based on external physical and/or chemical stimuli have been developed for a variety of emerging industrial and household applications [4,5,6,7]. Usually, these materials can change their shapes and translate chemical energy input into mechanical energy under environmental stimuli. Compared to conventional rigid actuators, soft actuators based on stimuli-responsive materials are composed of soft matter, i.e., polymers, elastomers or flexible composites. Their Young’s modulus is typically lower than 1 GPa, which is only 1/200 of the Young’s modulus of rigid actuators [8,9,10]. The excellent flexibility and adaptability of stimuli-responsive materials make soft actuators an ideal and critical element to be adopted in soft robots [11,12,13], artificial muscles [14,15,16,17,18], wearable haptics [19,20,21,22,23], and exoskeletons [24,25,26,27,28].

While great progress has been made in soft actuators, their implementation in real-world applications remains a challenge because of the limitations in activating conditions and some property flaws in current stimuli-responsive materials [29,30,31]. For example, electro-responsive soft actuators still retain complex circuits that need to be connected to an external power supply for effective actuation. These actuators have low degrees of freedom in motion as well as poor flexibility and adaptability that limit their applications in complex environments or small spaces. In particular, dielectric elastomer actuators often rely on high driving voltages of hundreds or thousands of volts for large deformations, which could bring serious safety concerns [32,33,34,35]. Similarly, pneumatic actuators require air pumps and control valves, increasing the volume and weight of the system [36,37,38]. 

Practical applications of stimuli-responsive materials in soft actuators are also hindered by material properties in terms of thermal expansion, magnetization characteristics, and photothermal conversion. For instance, electrothermal-responsive materials including shape memory alloys [39,40,41], shape memory polymers [42,43,44] and twisted fibers/yarns [45,46,47] are subjected to low thermal-responsive efficiency and low actuation frequency as a result of time-consuming heating and cooling cycles. Although magnetic soft materials have demonstrated their performance in some surgical applications [48,49,50,51,52,53], achieving high-precision and high-stability operation remains challenging, particularly in reprogrammable design and the fabrication of magnetization profiles. Photo-responsive materials employ ultraviolet (UV) irradiation to provide motion energy for actuators [54], but the response characteristics of photo-responsive materials are severely affected by their photothermal conversion efficiency. In addition, prolonged UV irradiation can damage most interacting objects, especially biological tissues and organs. The actuators that respond to a single stimulus often have a single deformation mode and relatively small deformation. To achieve more complex deformations, researchers have combined multiple driving mechanisms and smart materials to prepare some multi-responsive actuators [55,56,57,58].

Although many review articles have presented soft actuators and actuation materials, the actuation mechanism and its corresponding materials have not been comprehensively established, preventing researchers from entering this emerging field. In this review, we focused on presenting the actuation mechanism and development of biomimetic stimuli-responsive materials for soft actuators. Different principles of external stimuli in these responsive materials are first illustrated and discussed, including fluidic, electrical, thermal, magnetic, light and chemical stimuli. Then, state-of-the-art stimuli-responsive materials for soft actuators are summarized, including electroactive polymers, magnetic soft composites, hydrogels, liquid crystal elastomers, shape memory alloys and chemical-responsive materials. In addition, we also compare the mechanical properties of these stimuli-responsive materials. Finally, we discuss opportunities in the field of soft actuators and highlight the challenges that need to be addressed in order to achieve real-world applications of multifunctional artificial soft actuators.

## 2. Stimuli-Responsive Principles of Soft Actuators

Biomimetic stimuli-responsive soft actuators can be carefully created through selecting or synthesizing desired materials, designing functional structures, and establishing manufacture protocols [10]. By introducing external stimuli such as fluid pressure, electric fields, magnetic fields, thermal fields, illumination, and chemical substances in the design, biomimetic soft actuators can generate specific strains or deformations. Such deformation is achieved by converting multiple energy inputs into mechanical energy outputs, providing the necessary power. The promising attributes of elastomers/soft materials, such as their high flexibility, super-elasticity, viscoelasticity, and hysteresis characteristics, can be fully utilized in driving and controlling biomimetic soft actuators and further improving the overall performance of soft actuators.

### 2.1. Fluidic Stimuli

Fluidic actuation can be categorized into hydraulic actuation and pneumatic actuation. Hydraulic actuation is widely used in traditional industrial robotic arms/hands. Compared to pneumatic actuators, hydraulic actuators offer several advantages, including a large driving force, a high power-to-weight ratio, fast response speed, and low compressibility of the fluid medium [59,60]. However, pneumatic actuation is a more commonly used driving method in soft actuators/robots than hydraulic actuation, with two forms of actuation: positive-pressure actuation and negative-pressure actuation. Positive-pressure actuation involves filling the cavities of pneumatic actuators with gas to achieve bending, extending, twisting, and other deformations of soft actuators. Negative-pressure actuation, on the other hand, involves removing the gas inside to cause the cavities to shrink and deform, thereby causing the soft actuator to move [61].

**Figure 1:**
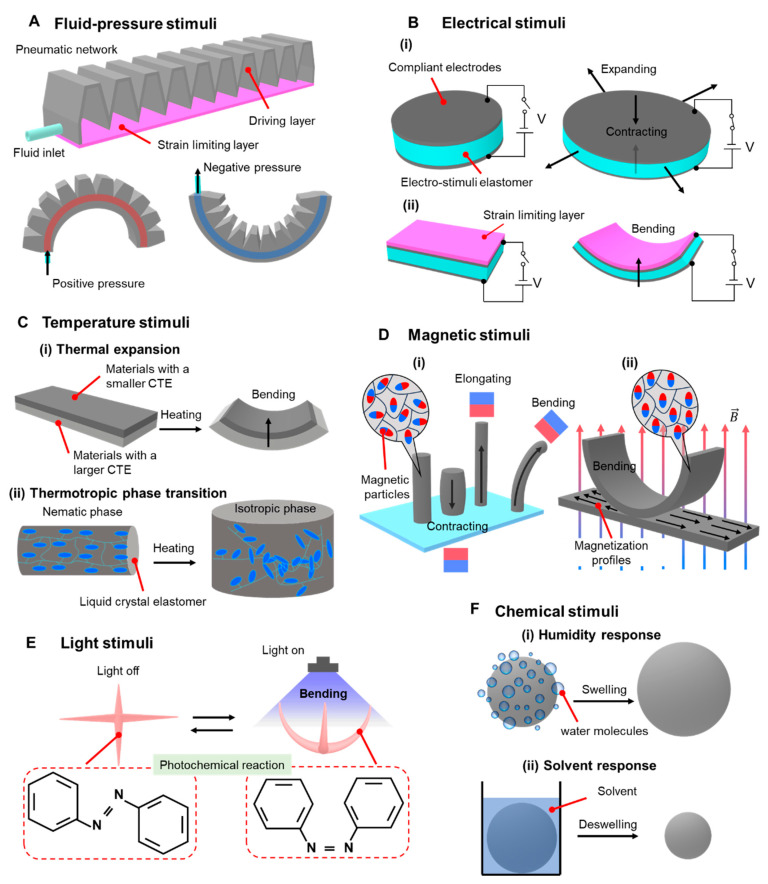
Stimuli-responsive principles of biomimetic soft actuators. (**A**) Pneumatic network actuators are actuated by positive-pressure or negative-pressure gas. (**B**–**F**) Actuation mechanisms triggered by external stimuli: electric fields (**B**), temperature (**C**), magnetic fields (**D**), light (**E**) and chemical stimuli (**F**).

Pneumatic networks (PNs) are a commonly used structure for pneumatic soft actuators, consisting of a driving layer and a strain limiting layer [37,38]. The driving layer is designed with multiple small cavity networks inside a flexible material, while the strain limiting layer is made of non-stretchable or highly elastic modulus materials. The driving layer and the strain limiting layer are sealed through bonding. When driven by positive-pressure gas, a PN actuator bends towards the side of the strain limiting layer. Conversely, when driven by negative-pressure gas, a PN actuator bends towards the side of the driving layer (Figure 1A). The bending performance of PN actuators can be easily improved by adjusting the morphology, the wall thickness of cavities, and the material characteristics.

### 2.2. Electrical Stimuli

Electrically driven soft actuators are commonly fabricated using electroactive polymers. These polymers are capable of undergoing macroscopic mechanical deformations when an electric field is applied to them [58,62]. One of the most prevalent electroactive polymers is the dielectric elastomer (DE). In a typical design of a DE actuator, as shown in Figure 1(Bi), the upper and lower surfaces of the DE film are coated with compliant electrodes, forming a parallel plate capacitor. When a high voltage is applied to these compliant electrodes, opposite charges are generated on both sides of the DE actuator, creating a Maxwell stress that induces the actuator to contract along its thickness direction and increase its area [63,64,65]. In order to achieve greater strain energy, DE actuators typically require a rigid frame and a pre-tensioning step [66]. Without pre-tensioning, DE actuators have a lower output force, a smaller strain, and a lower energy density, unless they incorporate multi-layer strain-stiffening elastomers [67] (Figure 1(Bii)). Electrically driven soft actuators offer advantages such as a high energy density, strong flexibility, a fast response rate, and large deformation. As a result, they are widely used in the design of soft robots.

### 2.3. Thermal Stimuli

Thermal-responsive soft actuators are primarily based on two types of materials: electrothermal responsive materials and photothermal responsive materials. Electrothermal responsive materials utilize an electrical current to generate Joule heating through thermal-resistive materials, causing the actuators to expand or contract. Common examples of electrothermal soft materials are shape memory alloys [39,40,41] and shape memory polymers [42,43,44,45,46,62]. These thermal-responsive soft actuators are designed to achieve improved performance output and exhibit complex and diverse deformations. To achieve these functions, several effective strategies have been explored, including material anisotropic realignment [68], the optimization of structural design [69], the patterning of material properties using advanced manufacturing techniques [70], mechanically pretreating the materials [45,46], and integrating multi-layer structures with different coefficients of thermal expansion (CTE) [71,72] (Figure 1(Ci)). 

Liquid crystal elastomers (LCEs) are widely used as photothermal-responsive materials due to their inherent anisotropy and ability to undergo reversible actuation with large anisotropic deformations (up to 500% strain). This deformation occurs when LCE materials are heated above a certain temperature, causing the prealigned crystalline phase to reorient from anisotropic to isotropic (Figure 1(Cii)). By coupling with the photomechanical effect, LCEs can achieve complex shape deformations [73]. However, LCE actuators suffer from a slow response speed and low driving efficiency, mainly due to the long heating and cooling cycles required [10].

### 2.4. Magnetic Stimuli

Soft actuators driven by external magnetic fields are produced by incorporating magnetic micro/nanoparticle fillers (neodymium iron boron and ferroferric oxide) into soft matrix materials (Ecoflex, polydimethylsiloxane, hydrogel). These magnetic soft actuators, as depicted in Figure 1(Di), exhibit deformations such as elongation, contraction, and bending when subjected to external magnetic fields [74]. Unidirectional magnetization technology, typically based on origami structures, is commonly employed to encode the internal magnetization profiles of these magnetic soft actuators. Magnetic soft actuators are firstly folded into predetermined shapes and then magnetized to saturation using a strong unidirectional magnetic field [75,76]. Consequently, magnetic soft actuators acquire magnetization profiles that align in parallel with the direction of the magnetization field. In recent advances in magnetic responsive materials, Pena-Francesch et al. synthesized a magnetic gel material, which may open up new opportunities for soft robotics, medical devices, and sustainable organic magnets [77].

When subjected to a spatially uniform actuation magnetic field, the magnetization profiles of magnetic soft actuators gradually align with the direction of the magnetic field (as shown in Figure 1(Dii)). Depending on the specific application, different magnetization profiles are required to achieve different motion modes. By simply folding the magnetic soft actuator into a new shape and magnetizing it again to saturation, new deformation information can be encoded into the actuator.

By modulating the parameters of the external magnetic field (such as amplitude, gradient, and direction) and the magnetization profiles, magnetic soft actuators can achieve multimodal locomotion in various complex environments [78]. They can also exhibit biomimetic movement and functions [79], as well as complex deformations [80,81]. The strength of magnetization, driving signals, and overall shapes of the filling materials play crucial roles in determining the deformation modes of magnetic soft actuators. Microscale magnetic soft robots are often designed for applications in enclosed spaces or clustered environments, particularly in interventional medical scenarios [82,83,84,85].

### 2.5. Light Stimuli

Photo-responsive actuation is advantageous for long-distance and non-contact actuation. Photo-responsive actuators can be controlled by manipulating the size, energy, and wavelength of light sources, allowing for selective response, local response, and spatiotemporal response in photo-responsive soft actuators. In comparison to magnetic soft actuators, light sources can be modulated with higher spatiotemporal resolution using optical choppers, lenses, and photomasks [86].

The working principles of photo-responsive actuators primarily rely on the mechanisms of the photothermal effect, photoelectric conversion, and photochemical reactions (Figure 1E). When exposed to light, these actuators can undergo macroscopic deformations and motions owing to their unique characteristics in various photo-responsive materials such as carbon-based materials, gel materials, and liquid crystal materials [87,88,89,90]. These materials exhibit specific features including shape memory [91,92], photothermal strain [93,94], photothermal adsorption and desorption [95,96,97,98], photothermal tension [99], photoisomerization [100,101,102] and photomagnetic properties [103,104], which enable their responsiveness to light stimuli. Currently, photo-responsive actuators mainly utilize photothermal expansion, photothermal adsorption or desorption, and the photothermal Marangoni effect [105,106] to achieve controlled and reversible deformations and motions. These mechanisms hold great potential for a wide range of applications.

### 2.6. Chemical Stimuli

Chemical-responsive actuation is a type of contact-responsive actuation method that involves direct contact between stimuli sources and soft actuators. This method allows for highly sensitive and rapid response speeds. Chemical substances are able to penetrate into the interior of soft actuators, leading to significant deformations. Some common examples of chemical stimuli sources include water, volatile solvents, salt solutions, and acid–base solutions. When these substances come into contact with soft actuators, they can induce various types of deformations and motions. This makes chemical-responsive actuation a versatile and powerful approach for a range of applications.

Humidity-responsive actuators are a type of contact-responsive actuation method that is triggered by water molecules. These actuators are made from materials that contain a high number of hydrophilic oxygen-containing functional groups which can reversibly adsorb or deadsorb water molecules [87]. This adsorption or deadsorption process leads to changes in the molecular gaps within the materials, resulting in the expansion or contraction of humidity-responsive actuators (Figure 1(Fi)). When the surrounding humidity increases, humidity-responsive actuators made from hydrophilic materials absorb moisture and expand. Conversely, when the surrounding humidity decreases, the actuators shrink as moisture detaches from the materials [107]. This reversible response to changes in humidity makes humidity-responsive actuators useful in various applications.

Solvent-responsive soft actuators are designed to selectively adsorb volatile chemical solvents such as acetone, ethanol, and tetrahydrofuran. These solvents can penetrate into the interior of the actuators and induce rapid and reversible deformation due to anisotropic volume changes [108,109,110] (Figure 1(Fii)). This property makes solvent-responsive actuators useful in applications where a specific solvent needs to be detected or controlled.

pH-responsive soft actuators are capable of sensing changes in pH values in the surrounding environment. These actuators are primarily designed for operation in liquid environments [111]. They can undergo structural changes, such as expansion or contraction, in response to variations in pH levels. This pH sensitivity enables these actuators to be used in applications where pH changes need to be monitored or utilized for actuation purposes.

## 3. Stimuli-Responsive Materials for Soft Actuators

The application advances of representative stimuli-responsive materials for soft actuators will be discussed in this section. These materials mainly include electroactive polymers, magnetic soft composites, hydrogels, liquid crystal elastomers, shape memory alloys and chemical-responsive materials. The physical properties and stimuli-responsive performance of these typical materials with distinct activation mechanisms are summarized in Table 1. We noticed that these materials have a Young’s modulus ranging from <100 kPa to >2 GPa, and that the response time varies from ~1 ms to >100 s depending on the stimulus method. In addition, the functioning durability also varied from <10 cycles to >10^5^ cycles. Based on their stimuli methods, we discuss each of these soft materials in the following.

### 3.1. Electroactive Polymers

Electroactive polymers (EAPs) are polymers that exhibit a high power density and remarkable mechanical compliance, making them suitable for simulating artificial muscles. These polymers can be categorized into two types based on their action mechanisms: ionic electroactive polymers and electronic electroactive polymers [129,130].

Ionic EAPs, including conductive ionic gels, conductive polymers and polymer-metal mixtures, have been used to fabricate soft actuators. These materials exhibit asymmetric volume expansion near the electrodes due to ion diffusion. The advantage of ionic EAPs is that they only require a low voltage of 1~2 V to generate a large deformation. However, maintaining constant deformation can be challenging due to the irreversible consumption of electrolytes [112,113,131].

**Figure 2:**
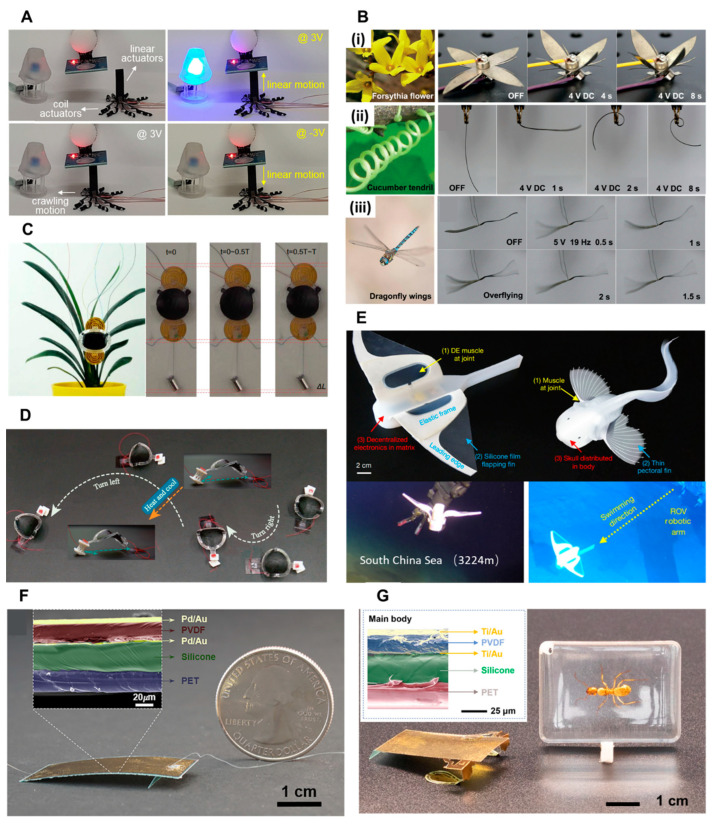
Biomimetic soft actuators based on electroactive polymers. (**A**) A spider robot based on ionic EAPs carries a linear actuator to switch on/off of LED bulbs (reprinted with permission from Ref. [114]. Copyright 2021, American Chemical Society). (**B**) Ionic EAP actuators mimicking: the blooming of forsythia flowers (**i**), the coiling behavior of cucumber tendrils (**ii**), and the high-frequency wing flapping of a dragonfly (**iii**) (reprinted with permission from Ref. [132]. Copyright 2019, John Wiley and Sons). (**C**) A soft wall-climbing robot based on DE artificial muscles (Reprinted with permission from Ref. [32]. Copyright 2018, the American Association for the Advancement of Science). (**D**) A soft crawling robot based on reconfigurable DE chiral grid feet (reprinted with permission from Ref. [140]. Copyright 2023, Springer Nature). (**E**) A wireless self-powered DE soft robot used for deep-sea exploration in the South China Sea and Mariana Trench (reprinted with permission from Ref. [141]. Copyright 2021, Springer Nature). (**F**) An insect-level soft robot based on a PVDF film (reprinted with permission from Ref. [151]. Copyright 2019, the American Association for the Advancement of Science). (**G**) A piezoelectric-driven soft robot with electrostatic footpads (reprinted with permission from Ref. [152]. Copyright 2019, the American Association for the Advancement of Science).

Researchers have made progress in developing advanced ionic EAP soft actuators. For example, Park et al. designed a five-layer structured soft actuator using ionic EAPs, where the porous intermediate layer improves the electromechanical performance and charging capacity [114]. This soft actuator can generate crimping, crawling, and bending behaviors under a low voltage. They also created a spider-shaped soft actuator that can move back and forth, demonstrating potential applications such as smart switches (Figure 2A). Ma et al. developed ionic soft actuators based on perfluorosulfonic acid with high-quality Pt electrodes using a chemical coating method assisted by isopropanol [132]. The Pt electrodes significantly enhance the driving performance of ionic actuators, enabling a low driving voltage of 1 V, a deforming amplitude of up to 35.3 mm, a response frequency of up to 10 Hz, and a bending degree of up to 596.2° s^−1^. These exceptional features allow the soft actuator to mimic the blooming of a forsythia flower, the coiling behavior of a cucumber tendril, and the high-frequency wing flapping of a dragonfly (Figure 2B).

Electronic EAP soft actuators are commonly designed as a sandwich structure, where the EAP materials are placed between two electrodes. These actuators typically require a high voltage input in order to generate a high mechanical energy density. When a high electric field is applied, the EAP materials undergo expansion in the area direction and contraction in the thickness direction, which is a result of Maxwell stress [133]. Among the various types of electronic EAPs, soft actuators made from dielectric elastomers (DEs) exhibit several advantages such as a higher energy density, faster response speed, better driving stability, and larger driving force [134]. However, conventional DE actuators often require a higher driving voltage due to their relatively low dielectric coefficient. Currently, extensive research efforts are focused on enhancing the dielectric constant of DEs and improving their dielectric breakdown strength. Additionally, reducing the elastic coefficient and driving voltage is also a key area of interest [135,136,137].

In the study conducted by Gu et al., a soft wall-climbing robot was developed using artificial muscles made from DEs. The robot demonstrated the ability to climb vertical wooden walls at a 90° angle while carrying a 10 g payload (Figure 2C). It was also capable of carrying cameras to capture videos in vertical tunnels, adjusting its body height to navigate through confined spaces and following labyrinth-like trajectories on a flat surface [32]. Although DE actuators exhibit a small driving force due to their biaxial strain response behavior, there are two commonly used methods to convert this behavior into linear motion. One approach involves stacking multiple layers of DE films, while the other involves folding a single layer into multiple layers of DE films [115,138,139]. Wang et al. proposed a soft crawling robot that utilized reconfigurable DEs with chiral grid feet (Figure 2D). This robot was capable of rapidly switching between forward, backward, and circular motions by adjusting the frequency of the input voltage. It also demonstrated the ability to reconstruct its structure under external temperature stimulation [140]. Li et al. developed a wireless self-powered soft robot using DEs that was inspired by a deep-sea fish called *Pseudolaris swirei*. This robot was designed for deep-sea exploration and was tested at depths of 10,900 m in the Mariana Trench and 3224 m in the South China Sea (Figure 2E). These tests confirmed that the robot exhibited excellent pressure resistance and swimming performance, showcasing its suitability for deep-sea exploration [141].

In recent years, 3D printing techniques, specifically direct ink writing, have been widely employed to fabricate DE actuators with complex structures that enable three-dimensional motion. For instance, Lewis et al. utilized 3D printing to create coaxial 3D fiber bundles and Janus-shaped coils. These coil actuators, when subjected to a voltage, bent towards the fiber side of the dielectric elastomer, enabling intricate and controllable driving behavior [116]. Additionally, Chortos et al. used 3D printing to develop a high-precision cross-shaped DE actuator capable of generating a 9% in-plane contraction [65]. Such advancements in 3D printing technology have significantly contributed to the development of DE actuators with complex structures and improved functionality.

Piezoelectric materials are also commonly employed in electrically driven soft actuators, enabling the conversion of electrical energy into mechanical force. Specifically, when a piezoelectric material is exposed to a specific electric field, it induces mechanical deformation or pressure in a particular direction. Soft actuators based on piezoelectric materials harness the inverse piezoelectric effect to generate force by converting electrical energy into mechanical force. Recently, piezoelectric actuators have attracted much attention in the field of soft robotics owing to their advantages of high accuracy, fast response, high driving frequency, and lightweight [142,143,144].

The piezoelectric coefficient of organic flexible piezoelectric materials is generally lower than that of conventical rigid piezoelectric ceramics. However, organic piezoelectric materials are still considered the preferred choice for piezoelectric actuators because of their remarkable flexibility and ease of preparation. These materials offer advantages in terms of conformability, weight, and the ability to be integrated into various shapes and structures, making them suitable for applications requiring soft and flexible actuation. Currently, several flexible piezoelectric materials, such as polyvinylidene fluoride (PVDF) [145,146], polyurea [147,148], and peptides [149,150], have been widely utilized in the development of soft actuators. For instance, Wu et al. [151] developed an insect-level soft robot using PVDF which runs at a very fast speed of up to 20 body lengths per second (Figure 2F). Furthermore, Liang et al. [152] further enhanced the functionality of soft robots by incorporating two electrostatic foot pads. This addition enabled the soft robot to achieve precise directional control. In their research, they utilized piezoelectric thin films as the main body, allowing the robot to navigate through mazes with flexibility (Figure 2G). Moreover, when equipped with gas sensors, this soft robot holds significant potential for detecting gas leaks.

Organic flexible piezoelectric materials have attracted significant interest in the realm of soft robotics owing to their exceptional electromechanical properties. Nevertheless, the limited deformation amplitude and high actuation voltage pose challenges to their widespread application. Consequently, future research on piezoelectric soft actuators will concentrate on developing wireless strategies and low-voltage piezoelectric materials. These advancements will enhance the performance and expand the potential applications of piezoelectric materials in soft actuators.

### 3.2. Magnetic Soft Composites

According to the magnetization characteristics of magnetic materials, there are three main types: soft magnetic materials, hard magnetic materials, and superparamagnetic materials. Soft magnetic materials, such as iron, nickel, and silicon-based ferroalloys, possess high magnetic susceptibility and relatively low residual magnetism and magnetic coercivity [74]. When it comes to constructing magnetic soft robots using isotropic soft magnetic materials, it is challenging to achieve gait motion or gripping operations. This is because isotropic spherical soft magnetic particles do not have a preferred magnetization direction. As a result, the magnetic moment they receive is always parallel to the external magnetic field, leading to zero overall torque within the soft magnetic composites.

**Figure 3:**
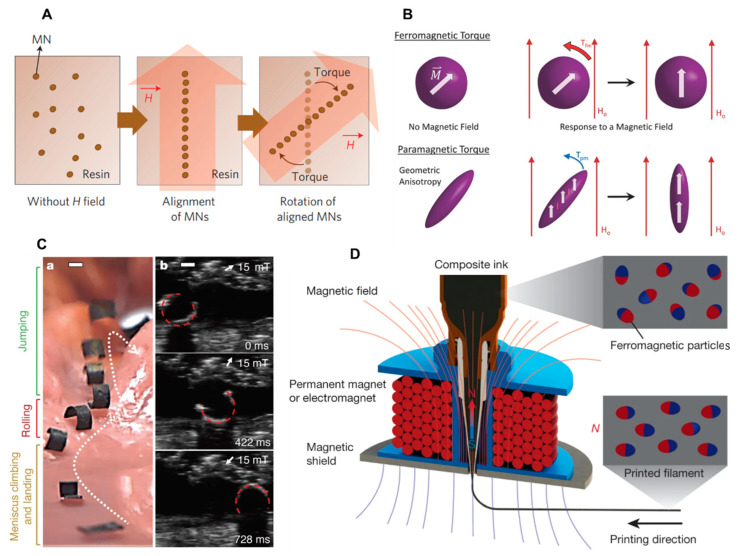
Magnetically responsive materials for biomimetic soft actuators. (**A**) Anisotropic soft magnetic particle chains built inside magnetic soft composites (reprinted with permission from Ref. [155]. Copyright 2011, Springer Nature). (**B**) Anisotropic soft magnetic materials such as rod-shaped and sheet-shaped employed to achieve local asymmetry (reprinted with permission from Ref. [156]. Copyright 2016, John Wiley and Sons). (**C**) Magnetic soft robot fabricated with hard magnetic materials navigating across a synthetic stomach phantom (reprinted with permission from Ref. [119]. Copyright 2018, Springer Nature). (**D**) Hard magnetic particles embedded in composite ink and reoriented by an applied magnetic field around the dispensing nozzle (reprinted with permission from Ref. [120]. Copyright 2018, Springer Nature).

Magnetic soft actuators made from soft magnetic soft composites can achieve simple deformations such as overall shortening, elongation, and bending by utilizing the magnetic attraction of nonuniform magnetic fields on magnetic particles [117,153,154]. In order to generate effective magnetic torque in soft magnetic materials, it is necessary to introduce some form of magnetic anisotropy to disrupt the symmetry of the soft magnetic material’s response to external magnetic fields. One approach is to build anisotropic magnetic particle chains inside magnetic soft composites to achieve overall anisotropy at the composite level [118,155] (Figure 3A). Another approach is to use anisotropic soft magnetic materials, such as rod-shaped [156,157] or sheet-shaped particles [158,159], to achieve local asymmetry at the magnetic particle level (Figure 3B). These strategies enable soft magnetic actuators to exhibit more complex and controllable motions.

Hard magnetic materials are known for their strong anti-demagnetization ability and high residual magnetism. Some commonly used hard magnetic materials include hexagonal ferrite barium (BaFe_12_O_19_) and hexagonal ferrite strontium (SrFe_12_O_19_), which have a magnetic coercivity of up to 300 kA/m. Additionally, materials like samarium cobalt (SmCo_5_, Sm_2_Co_17_) and neodymium iron boron (Nd_2_Fe_14_B) exhibit even higher magnetic coercivity, exceeding 1000 kA/m [74,160,161,162]. These materials are widely utilized for applications that require strong and stable magnetic properties.

Magnetic soft composites, which are created by incorporating hard magnetic particles into soft polymer matrix materials, possess both hard magnetic properties and exceptional mechanical flexibility. Once these composites are magnetized to saturation, the residual magnetism remains relatively constant, regardless of the driving magnetic fields within the range of their magnetic coercivity [74]. The magnetization profiles of hard magnetic soft composites can be specifically designed to achieve desired deformations, regardless of the shape and orientation of the hard magnetic particles [77] (Figure 3C). This capability allows for the development of impressive hard magnetic soft actuators that can undergo reconfigurable and multi-dimensional deformations. Advanced manufacturing technologies, such as 4D printing techniques, have been utilized to manufacture diverse magnetization profiles [120] (Figure 3D), enabling the creation of complex and customizable actuator designs [163,164]. These advancements in manufacturing techniques have expanded the possibilities for designing and fabricating innovative hard magnetic soft actuators with a wide range of deformations.

When the size of soft magnetic materials is reduced to a critical size, the magnetic coercivity starts to decrease rapidly until there is no residual magnetism or magnetic coercivity left. Such soft magnetic materials are referred to as superparamagnetic materials [66]. By incorporating chained superparamagnetic particles into magnetic soft composites, overall magnetic anisotropy can be achieved, making this an effective approach for manufacturing magnetic soft actuators [155,165,166]. However, the loading capacity of superparamagnetic materials in soft matrix materials is generally lower than that of pure soft or hard magnetic materials [167,168]. This is because superparamagnetic particles are prone to agglomeration due to van der Waals forces [169,170,171]. This limitation needs to be considered when designing and fabricating magnetic soft composites using superparamagnetic materials.

To enhance the dispersibility of magnetic nanoparticles, polymers are often employed to coat or functionalize them, resulting in the formation of core–shell structures. This approach enables intergranular electrostatic repulsion, which helps to balance out the van der Waals attraction between magnetic nanoparticles [172]. However, when compared with other types of magnetic soft composites, the magnetization profiles per unit volume within magnetic soft actuators based on superparamagnetic materials are lower. This is primarily due to the lower concentration of magnetic particles in these composites. As a result, the magnetic torques or forces generated by these actuators are smaller, resulting in less pronounced magnetic hardening, elongation, shortening, and bending effects.

### 3.3. Stimuli-Responsive Hydrogels and Liquid Crystal Elastomers

Hydrogels are polymer materials with three-dimensional cross-linked networks that are highly hydrated. They are known for their excellent biocompatibility and high water content. The mechanical properties and responsiveness of hydrogels can be adjusted over a wide range via incorporating various functionalities into photo-responsive biomimetic systems. These unique properties make hydrogels ideal for intelligent responsive materials in fields such as soft robotics, artificial muscles, and nanogenerators [173,174].

**Figure 4:**
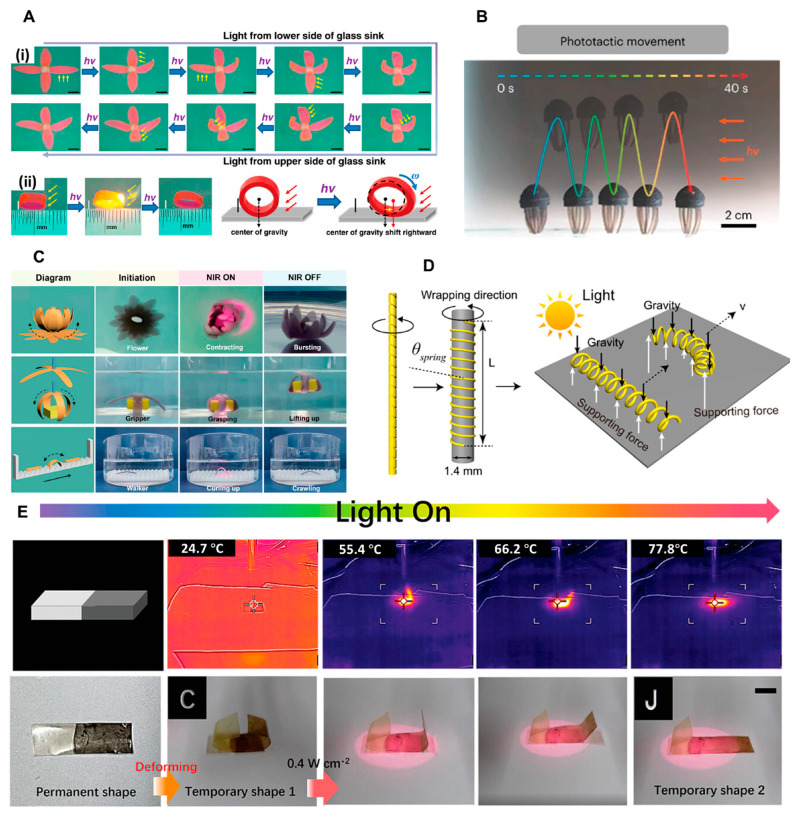
Photo-responsive materials for biomimetic soft actuators. (**A**) Localized photo-driven closing and blooming of biomimetic flowers (**i**), and photo-driven forward rolling of a wheel (**ii**) based on PU hydrogels (reprinted with permission from Ref. [121]. Copyright 2021, Elsevier Ltd.). (**B**) A hydrogel-based vehicle that can follow the direction of photon illumination (reprinted with permission from Ref. [179]. Copyright 2023, Springer Nature). (**C**) Opening and closing of flowers, soft grippers to grasp, lift and release goods, and a soft robot to crawl on the surface of a directional ratchet in water under near-infrared light (reprinted with permission from Ref. [180]. Copyright 2022, John Wiley and Sons). (**D**) A spring-type LCE fiber actuator capable of controlling the winding direction of twisted fibers (reprinted with permission from Ref. [192]. Copyright 2021, American Chemical Society). (**E**). An LCE soft actuator driven by local and sequential light with near-infrared photo-responsive polydopamine (reprinted with permission from Ref. [125]. Copyright 2023, John Wiley and Sons).

There are two main methods for fabricating hydrogel soft actuators. One approach involves creating a hydrogel actuator with a uniform surface and then applying uneven light stimuli to induce structural deformations. Owing to the photo-thermal effect, the temperature of the hydrogel actuator rises rapidly under light, leading to water vaporization in the irradiation area inside the hydrogel for actuations. For example, Xiang et al. developed a polyurethane (PU) hydrogel actuator with a uniform composition to achieve anisotropic deformations [121]. When exposed to light, the PU hydrogel actuator undergoes deformations such as expansion and bending. These deformations occur due to the decrease in cross-linking reactions and an increase in hydrophilicity on the illuminated side. By adjusting the position of the light source, the PU hydrogel actuator can exhibit curling, twisting, and folding motions (Figure 4A). This method allows for the precise control and manipulation of hydrogel actuators, enabling a wide range of motion possibilities for various applications.

Another approach is to construct hydrogel actuators with heterogeneous structures using various fabrication techniques such as stepwise synthesis, photolithography, 3D printing, ion-patterned crosslinking, magnetic/electric field induction and micro-assembly [122,123,175,176,177,178]. For instance, Hou et al. developed a hydrogel-based vehicle that can follow the direction of photon illumination and the internal direction to adjust the unconstrained fluid space [179]. By manipulating customized photothermal nanoparticles and micropores in polymer matrices, strong chemical mechanical deformation of soft materials was achieved (Figure 4B). Chen et al. developed a gradient hydrogel actuator with exceptional mechanical performance and durability [180]. This actuator enables several applications in underwater soft robotics for concept verification. Examples include simulating the opening and closing of flowers in water under near-infrared light, manufacturing light-driven soft grippers for grasping, lifting, and releasing objects, as well as achieving the photodynamic underwater movement of crawling robots on a directional ratchet surface (Figure 4C). These heterogeneous hydrogel structures offer advanced functionalities and expand the possibilities for developing innovative soft robotic systems with enhanced performance and adaptability.

Liquid crystal elastomers (LCEs) are a unique class of polymers that combine liquid crystal polyacrylates with the main or side chains of the polymer structure. They are characterized by excellent mechanical properties, self-healing, shape memory and fast reactions. By adjusting the orientation of liquid crystal molecules and the elastic entropy of polymers, soft actuators based on liquid crystal elastomers can achieve various deformation modes, such as expanding, contracting, curling and twisting [73,181,182,183,184]. In addition, adding photothermal materials to LCEs can convert light energy into thermal energy, thereby inducing LCE phase transition and producing deformations [185,186,187,188].

LCE actuators are mainly based on photochemical response and photothermal response [124,189,190,191]. Zhao et al. mixed liquid crystal Diels Alder networks (LCDANs) loaded with carbon nanotubes (CNTs) to prepare twisted fibers. Based on this, a spring-type CNT-LCDAN soft actuator further was manufactured by controlling the winding direction of the twisted fibers [192]. In their study, the CNT-LCDAN actuator was shown to achieve rolling with a speed of 22 mm/s in various environments (e.g., water and beach) under a continuous light stimulus (Figure 4D). In addition, Song et al. manufactured a photo-driven actuator by covalently crosslinking polyurethane (PU) into an LCE network (PULCN) [125]. In particular, by constructing a cross-linking density gradient on the thickness of the membrane, the PULCN actuator exhibits programmable initial shapes under a local and sequential near-infrared light (Figure 4E).

### 3.4. Shape Memory Alloys

Based on the deformation principle of shape memory alloys (SMAs), a series of SMA-driven worm-like actuators/robots have been developed to mimic the peristalsis, crawling and rolling of worms and geometries. The manufacturing methods of SMA-driven actuators is to embed SMA springs into soft polymers. SMA-driven actuators are based on thermoelastic martensitic transformation. This effect is caused by the phase transformation of the material at a certain temperature. By applying an electrical current to heat SMA springs for deformations, the polymer body passively deforms owing to deformations of the SMA springs. A robot’s wedge-shaped legs generate sufficient friction to obtain appropriate movement through the deformation of the body.

**Figure 5:**
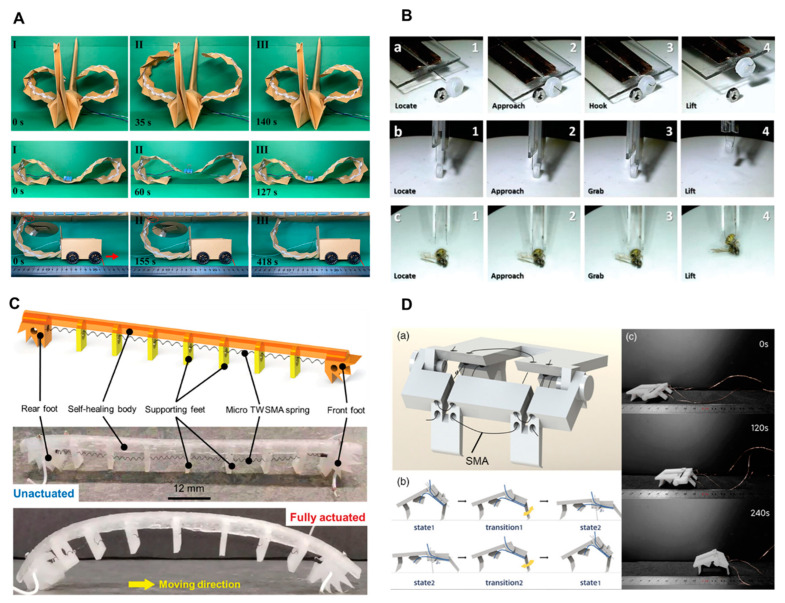
Shape memory alloys for biomimetic soft actuators. (**A**) Origami robots based on SMA wires (reprinted with permission from Ref. [126]. Copyright 2022, John Wiley and Sons). (**B**) Microscale SMA-based soft actuators fabricated via two-photon printing (reprinted with permission from Ref. [193]. Copyright 2020, John Wiley and Sons). (**C**) A flexible self-healing robot driven by miniature bidirectional SMA springs (reprinted with permission from Ref. [194]. Copyright 2024, John Wiley and Sons). (**D**) A non-adhesive origami SMA soft actuator: assembled robot structure (**a**), the links in a chain of periodic locomotion of the robot with two steady states and two transitions (**b**), photographic sequence of autonomous crawling on a smooth flat surface (**c**) (reprinted with permission from Ref. [195]. Copyright 2022, John Wiley and Sons).

Sangok et al. developed a mesh-like soft crawling robot (Meshworm) that mimics the peristaltic movement of earthworms [41]. In their research, the longitudinal SMA spring can shorten or bend the Meshworm, while the circumferential SMA spring contracts, which further causes Meshworm to elongate axially. Wu et al. [126] developed 3D-printed and origami robots that utilize SMA wires (Figure 5A). These robots were designed with “knitting-constraints” and were ridden using the SMA wires. In their experiments, the average speed achieved by the 3D-printed robot was 20 mm/min, while the origami robot had an average speed of 15 mm/min. This study demonstrates the use of SMA wires in robotic systems and shows the ability of the 3D-printed and origami robots to achieve controlled motion within a certain displacement range and at different speeds. Hyun-Taek et al. [193] proposed a small SMA soft composite actuator prepared via two-photon printing. As shown in Figure 5B, the deformation mode can be changed by changing the direction of the support lamination, and the driving can be controlled by the local electric heating effect of the carbon nanotube layer deposited inside the driver. This micro actuator can generate a force of 390 µN and achieve a bending angle of up to 80°, demonstrating the ability to lift and grip objects using single- and double-arm devices.

Liang et al. [194] developed a novel flexible self-healing robot driven by miniature bidirectional SMA springs (Figure 5C). This self-healing robot is driven by a new type of bidirectional shape memory alloy (TWSMA) spring actuator, with a crawling speed of 21.6 centimeters per minute, equivalent to 1.57 body lengths per minute. In addition, Zheng et al. [195] proposed a non-adhesive origami soft actuator (OSA) (Figure 5D). These OSA devices are composed of three key components: an SMA wire for generating driving force, a printing heater for thermal stimulation, and an elastic origami structure for providing restoring force. The printing heater used in this study has a length of 288 mm and is capable of maintaining a stable driving temperature of 105.1 °C when an applied voltage of 6.5 V is supplied. The printing heater plays a crucial role in activating the SMA wire by providing the necessary thermal energy for its shape memory effect. SMA actuators typically require a large electrical current for heating and a longer time for cooling, resulting in longer response times and significant hysteresis.

### 3.5. Chemical-Responsive Materials

Chemical-responsive materials used in biomimetic soft actuators mainly include humidity-responsive materials, solvent-responsive materials and pH-responsive materials. Humidity-responsive materials generally contain a large number of hydrophilic oxygen-containing functional groups that can reversibly adsorb or deadsorb water molecules, causing the molecular gaps of humidity-responsive materials to increase or decrease, thereby causing humidity-responsive soft actuators to expand or contract [107].

**Figure 6:**
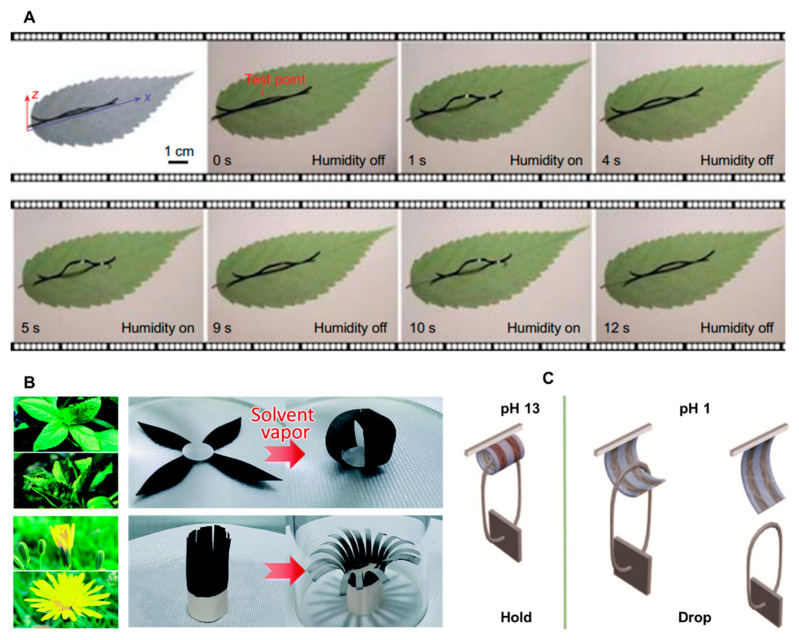
Chemical-responsive materials for biomimetic soft actuators. (**A**) A humidity-responsive GO/SU-8 actuator with a light weight (reprinted with permission from Ref. [127]. Copyright 2020, Oxford University Press). (**B**) Solvent-responsive PVDF/PPy actuators which can be used to mimic leaves and flower petals, respectively, using an acetone stimulus (reprinted with permission from Ref. [205]. Copyright 2021, the Royal Society). (**C**). A pH-responsive actuator which can curl into a tubular shape as the pH increases to 13 and is restored to a flattened state as the pH decreases to 1 (reprinted with permission from Ref. [128]. Copyright 2022, Elsevier Ltd.).

Materials that can generate a humidity response contain many hydrophilic functional groups. These functional groups can reversibly adsorb water molecules, causing the generation or removal of the molecular voids in the polymer matrix, thereby causing the material to expand or contract [196,197,198,199,200,201,202]. For example, Ma et al. [127] used soft lithography technology to prepare a double-layer structure of GO/SU-8 actuator. When the ambient humidity increased, the GO/SU-8 driver bent towards the SU-8 side. By adjusting the SU-8 pattern, the actuator can achieve controllable deformation (Figure 6A). Although this multi-layer actuator has a high deformation degree and a rapid response, it experiences problems regarding interlayer delamination. A feasible approach is to fill or modify other materials in the matrix material to create a gradient in the thickness direction of the matrix material. For instance, Han et al. utilized a magnetic field to create a concentration gradient of Fe_3_O_4_ in the thickness direction of the GO film [203], constructing a multi-responsive actuator. In addition, the GO-based actuator prepared by Qiu et al. has different surface morphologies on both sides. When the humidity increases, the GO-based actuator can bend towards the flat side [204].

Solvent-responsive materials used for soft actuators include liquid crystal polymers, hydrogels and semi-crystalline polymers [108,109,110]. For example, Yim et al. [205] polymerized polypyrrole (PPy) on hydrophobic PVDF membranes via gas-phase polymerization to prepare solvent-responsive actuators. As shown in Figure 6B, these solvent-responsive PVDF/PPy actuators can be used to mimic leaves and flower petals, respectively, under an acetone stimulus. In addition, Wang et al. [206] prepared single-layer asymmetric PVDF actuators using vacuum hot pressing technology. Under an acetone stimulus, directional and asymmetric expansion can be generated, and biomimetic behaviors such as entanglement and jumping can be achieved.

PH-responsive materials used to prepare actuators have ionizable groups (amine, pyridine and carboxyl) [111,207]. For example, polyacrylic acid (PAAc) contains a large number of carboxyl groups. When the environmental pH increases, the PAAc colloids expand owing to the increase in ionization of the PAAc chain. On the contrary, when the environmental pH decreases, the PAAc colloids contract [208]. Based on PAAc materials, Cho et al. [128] used electrospinning technology to fabricate a pH-responsive actuator with a bilayer structure. As shown in Figure 6C, the pH-responsive actuator curled into a tubular shape as the pH increased to 13. The pH-responsive actuator returned to a flattened state as the pH returned to 1. In addition, polyethylene glycol fatty acid ester patterns were selectively modified on the surface to effectively control the deformation direction of the pH-responsive actuator.

### 3.6. Multiple Stimuli Responsive Composites

In order to achieve more complex deformation, multiple single-stimulus-responsive materials have recently been combined to prepare multi-stimuli-responsive actuators [55,56,57,58]. These multi-stimuli-responsive actuators can achieve a series of programmable shape deformations, such as curling, spiraling, self-folding, and other complex three-dimensional shapes. With the synergistic effect of multiple stimuli, they can simultaneously complete complex operations including grasping, biomimetic crawling, and weightlifting. For example, Kumpfer et al. [209] reported a covalently photo-cross-linked metallo-supramolecular polymer with thermal, photo, and solvent responses. In this polymer, the covalently photo-crosslinked networks support the initial shape, while the hard chain segments of the metal ligand act as reversible phases to fix or release their temporary shapes. In this way, any stimulus (light, heat, or chemicals) that can disrupt this phase can be used to create a temporary shape and to induce its recovery to the predesigned permanent shape. By varying the cross-link densities of the soft-phase materials, this polymer can achieve initial strain fixing greater than 80% and strain recovery over 95%. In addition, Wang et al. [210] synthesized a block copolymer containing polyurethane and polymethyl methacrylate through phase separation. By adjusting the mass ratio of polyurethane and polymethyl methacrylate, the block copolymer exhibited thermal-, water-, and pH-induced shape memory effects with a temperature response range of 0–100 °C, a swelling degree in water up to 70%, as well as rapidly responding to 0.1 mol/L NaOH solution.

Moreover, Dong et al. [211] directly modified GO film with a top wax layer and fabricated GO/wax actuators with thermal- and humidity-responsive functions. Heating can cause the GO/wax actuator to bend towards the GO side, because the thermal expansion coefficient of wax is significantly higher than that of GO. Increased humidity can cause the GO layer to absorb water molecules and expand. Because the volume of the wax layer remains relatively unchanged, and the GO/wax actuator bends towards the wax layer. In this way, by tuning temperature and humidity, the actuator can bend in different directions with different deformation rates.

Zhang et al. [212] prepared a composite material responsive to thermal, electrical, and photo stimuli. In their study, graphene oxide was directly reduced into graphene in N-methyl-pyrrolidone with the assisted dispersion of vapor-grown carbon nanofibers (VGCF). The VGCF served as a stabilizer by adsorbing VGCF onto graphene through π–π interactions. Subsequently, the as-prepared VGCF-graphene (VGCF-G) was incorporated into a bio-based polyester (BE) forming BE/VGCF-G composites via solution blending. The binary combination of VGCF and graphene showed remarkable synergistic effects in improving the electrical conductivity and mechanical properties. Because of the excellent electrical conductivity and photothermal property of VGCF and graphene, BE/VGCF–G composites demonstrated electro-activated and infrared-triggered shape memory.

## 4. Conclusions and Outlook

In this review, we summarized different stimulus methods which cause responsive soft materials to regulate deformation and to form soft actuators. In addition, the underlying mechanisms to cause stimulus responses for different actuator materials were also introduced, including fluidic, electrical, thermal, magnetic, photonic, and chemical stimuli. Corresponding to each stimulus, we further established 6 types of stimuli-responsive soft matter, and discussed >20 different materials used as soft actuators. Among them, various soft robotics were introduced to demonstrate the promising impact of such materials, from climbing robotic worms [32] to swimming artificial jellyfish [179] and skates [141], to drug-delivery smart pill wraps [77]. Although stimuli-responsive materials in soft actuators are recognized as the primary element for next-generation robotics, there are great challenges to be faced in this field before they can be implemented in current robotic systems.

The precise control of stimuli-responsive materials on their actuation performance is extremely challenging, because most of them are hyper-elastomers with strong material nonlinearity, making it difficult to accurately establish their kinematic and dynamic mathematical models. Furthermore, most stimuli-responsive materials have significant response hysteresis, which seriously influences the control signal. Notably, animals accomplish complex motion through a multi-sensing system with rigid–soft synergistic actuators. Therefore, the optimized combination of soft and hard actuators through artificial intelligence tools could be one of the most valuable directions in addressing the precision control of actuation.

Increasing motion and energy-transfer efficiency in soft actuators is currently the most heavily invested research direction. It requires complex structural designs that span multiple scales and dimensions. This complexity poses challenges in terms of manufacturing. One key requirement for these manufacturing technologies is high precision, in order to ensure the desired functionality of soft actuators. Another important consideration is economic feasibility. High precision requirements would require manufacturing technologies like photolithography, which is expensive and not scalable. Therefore, high-precision manufacturing methods need to be coupled in the design consideration of soft actuators with optimizing processes, minimizing material waste, and streamlining production workflows.

Another challenge is to develop biocompatible stimuli-responsive materials, as this is crucial for biomedical use. Traditionally, non-toxic materials are regulated to be free from heavy metals, harmful chemicals, or any components that can pose a risk to human health or the environment. For materials used in humans, no toxicity is not sufficient. Allergic reactions and other adverse effects also need to be completely avoided, particularly when these devices are implanted, used in medical procedures, or interact with living organisms. With the increasing understanding of the harm caused by micro-plastics [213], biodegradability and biocompatibility are not only an important consideration for the environmental impact, but also for long-term implants, which cannot or are difficult to remove from the body. Biocompatible stimuli-responsive materials can naturally break down over time and be absorbed or eliminated by the body without leaving any harmful residues and without being attacked by the immune system. This property is particularly important for implantable devices or those used in drug delivery systems where the actuator may need to be adopted by the human body and degrade after fulfilling its intended purpose. By developing biocompatible stimuli-responsive materials, we can expand the application of soft actuators to broader biomedical applications.

More importantly, the durability of soft actuation materials is another issue that hinders the implementation to industrial robotic systems. The fatigue of a material affects its total actuation cycles, and therefore it has been heavily investigated in recent years [214]. Other factors that have been paid less attention include the mechanical strength [215] and anti-fouling capability [216], which could be the next focal point to explore.

Currently, the widespread deployment and acceptance of soft actuators are still rare in the commercial robotics ecosystem. Before addressing the aforementioned challenges, researchers in the fields of materials science, computer science, biology, and mechanical engineering involved in soft robotics need to collaboratively work together and seek innovative solutions to create satisfying soft stimuli-responsive materials for soft actuators implemented in industrial robotic systems.

## Figures and Tables

**Table 1:** Performance comparison of typical stimuli-responsive materials for soft actuators.

Materials	Stimulus Sources	Response Time	Mechanical Properties	Crosslinking Methods	Durability	Refs
Ecoflex 00-30	Fluidic stimuli	~50 ms	*E* = ~0.1 MPa	Thermal curing	1 × 10^5^ cycles	[37]
IPMCs	Electrical stimuli	~20–90 s	NA	Ionic crosslinking	NA	[112]
IGMN	Electrical stimuli	~50 s	*E* = ~0.35 MPa	Ionic crosslinking	7.6 × 10^4^ cycles	[113]
PEDGA, PEDOT: PSS	Electrical stimuli	~0.8 s	*E* = ~11–15 MPa	Ionic crosslinking	5 × 10^3^ cycles	[114]
PDMS	Electrical stimuli	~1.67 ms	*E* = ~4 MPa	Thermal curing	NA	[35]
PDMS	Electrical stimuli	5 ms	NA	Thermal curing	5 × 10^4^ cycles	[115]
FSNPs, Ecoflex 00-30	Electrical stimuli	1.43 ms	*G* = 27–979 kPa	Thermal curing	2.6 × 10^5^ cycles	[116]
Fe_3_O_4_ nanoparticles, Fe-alginate, PAAm	Magnetic stimuli	NA	*τ* = 200–1000 kPa	Ionic crosslinking	NA	[117]
Carbonyl iron microparticles, TPU	Magnetic stimuli	25 ms	NA	Thermal curing	NA	[118]
NdFeB microparticles, Ecoflex 00-10	Magnetic stimuli	NA	*E* = ~78.6 ± 4.8 kPa	Thermal curing	NA	[119]
Iron microparticles, PDMS	Magnetic stimuli	35.8 ms	*E* = ~2 MPa	Thermal curing	5 × 10^3^ cycles	[78]
NdFeB, FSNPs, Ecoflex 00-30 Part B, SE 1700	Magnetic stimuli	~0.1 s	*G* = 330 kPa	Thermal curing	NA	[120]
PEG_1000_, HDI, HABI	Light stimuli	~35 s	*τ* = 2.88 MPa	Light-crosslinking	500 cycles	[121]
BN, AlN, Si_3_N_4_, NIPAM	Light stimuli	~30 s	*E* = 9.71 ± 0.06 kPa	Photopolymerization	>10 cycles	[122]
PNIPAAM, AuNPs, rGO	Light stimuli	<1 s	*τ* = 8–18 kPa	Photopolymerization	NA	[123]
RM 257, HDT	Light stimuli	<0.2 s	*τ* = 3–4 MPa	Light-crosslinking	1 × 10^6^ cycles	[124]
PLCMs, MDI, HEMA	Light stimuli	4–8 s	*E* = ~11–20 MPa	Light-crosslinking	NA	[125]
Ni, Cr, SMP	Electrothermal stimuli	20 s	*E* = ~3.33–125.65 MPa	NA	NA	[126]
Graphene oxide, SU-8	Humidity stimuli	~22–26 s	*τ* = ~30–90 MPa	Photopolymerization	>500 cycles	[127]
PVDF, PPy	Solvent stimuli	3.1–9.2 s	*E* = ~2.53 GPa	Thermal curing	NA	[127]
PAAc	pH stimuli	~120 s	*E* = ~300 MPa	Photopolymerization	>10 cycles	[128]

*E*, Young’s modulus; *G*, shear Modulus; *τ*, ultimate stress; NA, not applicable; Ecoflex, a silicone rubber; PDMS, polydimethylsiloxane; IPMCs, ionic polymer–metal composites; IGMN, ionic gel/metal nanocomposite; PEDGA, poly(ethylene glycol) diacrylate; PEDOT:PSS, poly(3,4-ethylenedioxythio-phene)-poly(styrenesulfonate); DEs, dielectric elastomers; FSNPs, fumed silica nanoparticles; PAAm, polyacrylamide; TPU, thermoplastic polyurethane; NdFeB, neodymium–iron–boron; SE 1700, a silicone-based material; PEG_1000_, polyethylene glycol; HDI, hexamethylene diisocyanate; HABI, a tetrahydroxy functionalized crosslinker; BN, boron nitride; AlN, aluminum nitride; Si_3_N_4_, silicon nitride; NIPAM, n-isopropylacrylamide; PNIPAAM, poly(N-isopropylacrylamide); AuNPs, gold nanoparticles; rGO, reduced graphene oxides; RM 257, a liquid crystal mesogen; HDT, hexane dithiol; PLCMs, polymerizable liquid crystal monomers; MDI, diphenylmethane diisocyanate; HEMA, 2-Hydroxyethyl acrylate; SMP, shape memory polymer; SU-8, a type of photoresist; PVDF, polyvinylidene difluoride; PPy, polypyrrole; PAAc, Poly(acrylic acid).
